# ATP-Binding Cassette Transporter VcaM from *Vibrio cholerae* is Dependent on the Outer Membrane Factor Family for Its Function

**DOI:** 10.3390/ijms19041000

**Published:** 2018-03-27

**Authors:** Wen-Jung Lu, Hsuan-Ju Lin, Thamarai K. Janganan, Cheng-Yi Li, Wei-Chiang Chin, Vassiliy N. Bavro, Hong-Ting Victor Lin

**Affiliations:** 1 Department of Food Science, National Taiwan Ocean University, No. 2, Pei-Ning Road, Keelung 202, Taiwan; miss350100@gmail.com (W.-J.L.); angel810801@gmail.com (H.-J.L.); hicatex2003@hotmail.com (C.-Y.L.); jackie0522@livemail.tw (W.-C.C.); 2 School of Life Sciences, University of Bedfordshire, University Square, Luton LU1 3JU, UK; thamarai.janganan@beds.ac.uk; 3 School of Biological Sciences, University of Essex, Wivenhoe Park, Colchester CO4 3SQ, UK; 4 Center of Excellence for the Oceans, National Taiwan Ocean University, No. 2, Pei-Ning Road, Keelung 202, Taiwan

**Keywords:** ATP-binding cassette transporter, VcaM, *V. cholerae*, TolC, multidrug efflux pumps

## Abstract

*Vibrio cholerae* ATP-binding cassette transporter VcaM (*V. cholerae* ABC multidrug resistance pump) has previously been shown to confer resistance to a variety of medically important drugs. In this study, we set to analyse its properties both in vitro in detergent-solubilised state and in vivo to differentiate its dependency on auxiliary proteins for its function. We report the first detailed kinetic parameters of purified VcaM and the rate of phosphate (Pi) production. To determine the possible functional dependencies of VcaM on the tripartite efflux pumps we then utilized different *E. coli* strains lacking the principal secondary transporter AcrB (Acriflavine resistance protein), as well as cells lacking the outer membrane factor (OMF) TolC (Tolerance to colicins). Consistent with the ATPase function of VcaM we found it to be susceptible to sodium orthovanadate (NaOV), however, we also found a clear dependency of VcaM function on TolC. Inhibitors targeting secondary active transporters had no effects on either VcaM-conferred resistance or Hoechst 33342 accumulation, suggesting that VcaM might be capable of engaging with the TolC-channel without periplasmic mediation by additional transporters. Our findings are indicative of VcaM being capable of a one-step substrate translocation from cytosol to extracellular space utilising the TolC-channel, making it the only multidrug ABC-transporter outside of the MacB-family with demonstrable TolC-dependency.

## 1. Introduction

*Vibrio cholerae* is a Gram-negative non-invasive enteric pathogen and the causative agent of cholera—a severe diarrheal disease [1]. The proliferation of *V. cholerae* has been linked to plankton density in water, the chitin of which can be used by *V. cholerae* as carbon and nitrogen sources [2]. *V. cholerae* has over 200 serotypes based on the cell-surface O-antigens, with so far, only O1 and O139 serotypes being identified as cause of epidemic cholera. Cholera has been categorized as one of the re-emerging infections threatening many developing countries. While fluid replacement remains the most important component of therapy, antibiotic treatment with tetracyclines, fluoroquinolones and macrolides is central for limiting morbidity and mortality by inhibition of the growth of *V. cholerae* [3,4].

*V. cholerae* utilises a formidable array of antibiotic resistance mechanisms including chromosomal mutations, exchange of conjugative plasmids, self-transmissible chromosomally integrating SXT-elements and multidrug transporters [4,5]. *V. cholerae* has been shown to develop resistance to a broad range of frontline antibiotics including tetracycline, macrolides and fluoroquinolones [4]. Multidrug efflux pumps and transporters provide a first line of defence allowing development of additional resistance mechanisms and, thus, understanding their function is critical for addressing it. To date, numerous multidrug transporters have been identified and investigated in *V. cholerae*, e.g., VexAB and VexCD from Resistance-Nodulation-Division (RND) family [6,7]. Colmer, et al. [8] indicated that the VceB transporter, a homologue of *E. coli* EmrB, belonging to the Major Facilitator Super family (MFS) forms a tripartite system with membrane protein VceA and an outer membrane factor (OMF) VceC [9]. The Multidrug And Toxic Compound Extrusion (MATE) family transporters VcmA [10], NorM [11] and VcrM [12], driven by electrochemical potential of Na^+^, have been reported. In addition, Huda, et al. [13] reported the primary active ATP-binding cassette (ABC) transporter VcaM (GenBank Q93GU0) conferring drug resistance. Several different groups of active transporters (including RND, ABC and the MFS families) require a member of TolC (OMF) family form functional tripartite efflux pumps [14]. In *E. coli*, for example, TolC is shared amongst the RND-transporter-based efflux pump AcrAB-TolC, ABC-transporter efflux pump MacAB and MFS-transporter based efflux pump EmrAB all of which have been known to transport antimicrobial agents [15,16,17,18]. TolC family members are present in most Gram-negative bacteria, such as MtrE (Multiple transferable resistance) in *Neisseria gonnorhoeae* [19,20,21], TolC and VceC in *V. cholerae*, and OprM, OprJ and OprN (outer membrane protein responsible for multiple-drug resistance) in *P. aeruginosa* [9,22,23]. TolC orhologues in *V. cholerae* have been demonstrated to be essential for transport of bile acids, erythromycin, SDS and other xenobiotic [22]. In addition, TolC orthologues are also involved in the ABC-transporter-based type 1 secretion systems (T1SS) such as RTX (Repeats-in-toxins) toxin secretion in *V. cholerae* [24] and the well characterized HlyBD-TolC in *E.coli* [25].

One puzzling issue with the potential tripartite pump which may contain VcaM in *V. cholerae* is the lack of obvious periplasmic adapter proteins (PAPs) associated with the VcaM locus. However, our sequence analysis (summarized in Table 1 below) reveals a number of potential candidate PAPs which can plausibly associate with VcaM to form a functional pump based on their homology to established tripartite pump systems in *E. coli*. As shown in Table 1, the PAPs AcrA and AcrD of *E. coli* share a sequence identity of 36% with *V. cholerae* homolog MexC. *E. coli* MacA shared a sequence identity of 38% with *V. cholerae* hemolysin D (HlyD), a PAP of the TISS (Type I secretion system). *E. coli* EmrA and EmrK shared a sequence identity of 39 and 40% with *V. cholerae* VceA, respectively.

VcaM has been reported to have an effect on drug susceptibility and dye accumulation in vivo [13], but so far it has not been overexpressed and purified in a detergent-solubilized state. Thus, the kinetic parameters of VcaM have not been determined in vitro in detail. In this study, we present the first direct kinetic parameters of VcaM from *V. cholerae* based on an in vitro detergent-solubilised form and, by using a range of different *E. coli* genetic knock-out strains, demonstrate for the first time its functional dependency in vivo on the OMF TolC. Furthermore, our data clearly demonstrates that VcaM is not dependent on secondary RND transporters for its efflux function suggesting that it is capable of directly bridging the TolC channel without the substrate being released as a periplasmic transport intermediate.

## 2. Results and Discussion

### 2.1. Overexpression and Purification of VcaM in E. coli

**Figure 1:**
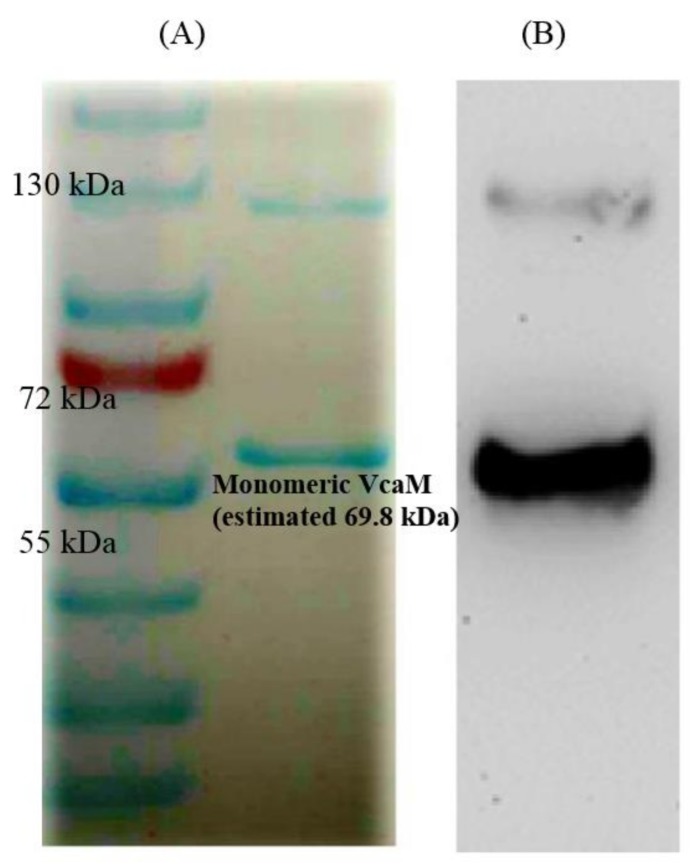
The (**A**) Sodium dodecyl sulfate-polyacrylamide gel electrophoresis (SDS-PAGE) and (**B**) Western blot of *V. cholerae* VcaM. The 10% SDS-PAGE showed the purified fraction of monomeric VcaM (right lane, between 55 and 72 kDa) and dimeric VcaM (right lane, slightly below 130 kDa). The Western blot was performed with a mouse anti-histidine tag primary antibody and goat anti-mouse IgG-AP conjugate as the second antibody.

In order to determine the kinetic parameters of the putative ATPase transporter VcaM from *V. cholerae* (GenBank Q93GU0), the *vcaM* gene was amplified and cloned onto pET21a vector to generate the plasmid pET21a-*vcaM*. The plasmid was then transformed into *E. coli* C43 (DE3) cells. VcaM expression was induced with 0.2 mM IPTG, and purified by using the nickel affinity column. The overexpression of VcaM in C43 (DE3) was confirmed by using SDS-PAGE and Western blot (Figure 1).

SDS-PAGE (sodium dodecyl sulfate polyacrylamide gel electrophoresis) revealed major protein bands with the apparent molecular weight of approx 65 kDa and 130 kDa respectively (Figure 1). The band at 65 kDa molecular weight was confirmed to be the VcaM protein and is consistent with the predicted size of 69.8 kDa of the *vcaM* gene product. The protein band at 130 kDa is consistent with a dimeric form of the VcaM, which is typical of the ABC-transporters with split NBD-domains (e.g., previously reported MacB exhibits similar behaviour [18,32]). The protein band of dimeric VcaM was resistant to boiling in the presence of reducing agent, suggesting that the VcaM dimers might have been stabilized by non-disulphide covalent bonds. Consistently, Western blot using HisProbe confirms this interpretation (Figure 1).

### 2.2. Kinetic Parameters of ATP Hydrolysis by VcaM

**Figure 2:**
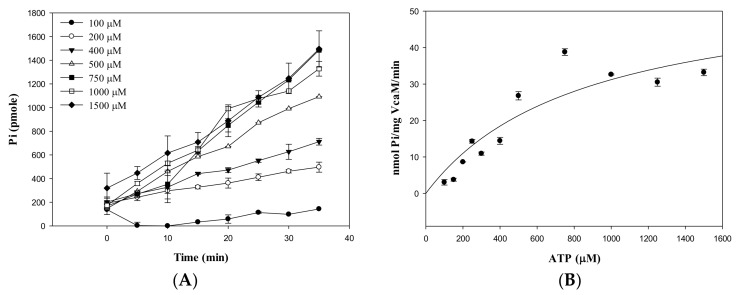
The Adenosine triphosphatase (ATPase) activity of VcaM. (**A**) Pi generation by VcaM on various concentration of ATP (B) The steady-state rate of Pi production by VcaM. The ATPase activity was measured by malachite green assay using 0.5 mM VcaM in detergent-solubilised state.

Well-characterised ABC transporters, such as MacB from *E. coli*, displayed a basal ATPase activity in detergent micelles. VcaM has been reported to have an effect on the drug susceptibility and dye accumulation in vivo [13], but the kinetic parameters of VcaM have not been determined in vitro in detail. The kinetic parameters of VcaM in its detergent-solubilized state were measured using ATPase assay based on a Malachite green system, which is based on the quantification of a green-coloured complex formed as a result of inorganic phosphate reacting with Malachite Green and ammonium molybdate. For that, we investigated the hydrolysis of different concentrations of ATP by the purified VcaM (Figure 2A). The results show that ATP hydrolysis increases in a concentration-dependent manner. From the plots, we derived the kinetic parameters of purified VcaM from affinity chromatography which gave a V_max_ of 58 nmol/mg^−1^ min^−1^, a K_m_ value of 852 µM and a K_cat_ of 0.07 s^−1^ in a steady state rate of Pi production (Figure 2B), which is the first time that the detailed kinetic parameters of detergent-solubilized VcaM have been reported.

This level of activity is comparable to the previously reported ATPase activity of purified MacB, which has a K_m_ value of 910 µM and a K_cat_ of 0.17 s^−1^ [32]. It was previously reported that Sav1866 from *Staphylococcus aureus* exhibited a V_max_ of 63 nmol Pi/mg^−1^min^−1^ although the K_m_ value for ATP was not determined [33], and the McjD from *Escherichia coli* gave a K_m_ of 169.3 ± 6.7 μM and a V_max_ of 44.4 ± 0.5 nmol min^−1^ mg protein^−1^ [34].

### 2.3. VcaM Efflux in E. coli Depends on the OMF TolC.

**Figure 3:**
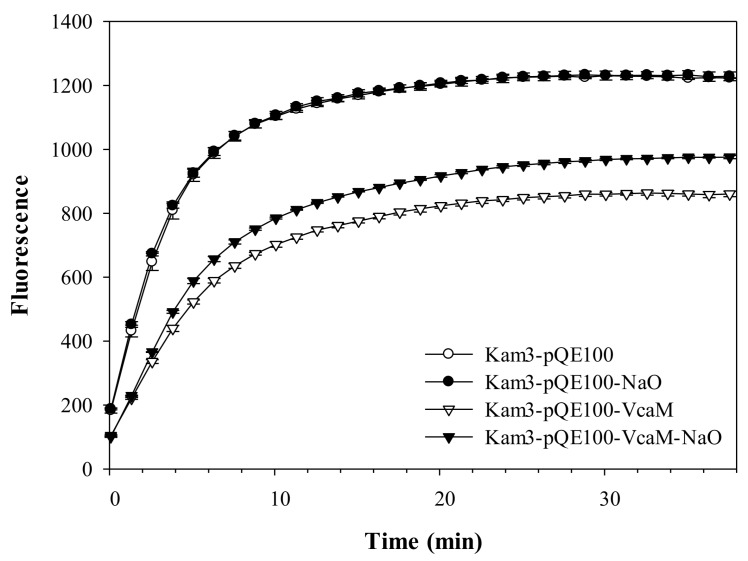
Drug accumulation of H33342 in *E. coli* Kam3 overexpressing VcaM in the presence of ABC transporter inhibitor NaOV. H33342 was incubated for 38 min in the presence of 40 μg/mL NaOV.

The OMF TolC is an essential part of many tripartite efflux pumps, but to date, it has not been shown to associate directly with any other ABC-transporter-based efflux pumps apart from the highly-specialised MacB-family [35]. Indeed, the majority of secondary and ABC-transporters appear to pump their cargoes into the periplasmic space, from where they are picked up by broad-specificity RND transporters such as AcrB and siphoned out of the cell using the OMF TolC as a universal duct [36]. For eample, in *Salmonella*, at least nine different efflux systems converge on TolC [37]. Based on these observations, in orderto analyse the potential mechanism of the drug efflux by VcaM we chose to use the drug-sensitive *E. coli* strains Kam3 (Δ*acrB*) and TG1 cells (Δ*tolC*) in efflux studies to allow us to decouple the two stages of drug transport described above and to establish whether the substrate is being released into the periplasm to be picked up by RND-pumps or proceeds directly to TolC. We reasoned that if VcaM operates in an RND-dependent fashion we should see a prominent activity contribution in a Hoechst 33342 accumulation assay, a dye which becomes greatly fluorescent when it binds to DNA and lipid membranes. We used time-resolved Hoechst H33342 fluorescence to monitor whether VcaM is (a) able to provide increased efflux function in a heterologous expression system, (b) requires secondary RND transporters for it and, (c) whether its function is dependent on the duct of the OMF TolC to efflux substrates. Although effective efflux by heterologously expressed VcaM has been reported already [13], we validated its activity in cells lacking the secondary transporter AcrB [Kam3 cells (Δ*acrB*)] in the presence and absence of the ATPase inhibitor sodium orthovanadate (NaOV) (Figure 3). This allows for a higher sensitivity of the assay as it removes the function of the AcrB, which is the dominant efflux pump in *E. coli* often masking the activity of the minor transporters.

First, in order to confirm that the efflux depends on the ATPase activity of VcaM, the accumulation of H33342 was performed in the Kam3 cells (Δ*acrB*) harbouring plasmid encoding VcaM (Kam3-pQE-VcaM). The efflux of H33342 was higher in cells expressing VcaM than the cells harbouring control plasmid, confirming that VcaM is active and capable of H33342 substrate expulsion. Accordingly, addition of NaOV to cells expressing VcaM caused a notable increase of H33342 accumulation. Addition of vanadate, a phosphate analogue, in the presence of ATP, resulted in stable trapping of MgADP·Vi in one of the ATPase catalytic sites [38], and this data indicated that the activity of VcaM was responsible for the majority of H33342 efflux (Figure 3). 

The fact that the VcaM seems to be readily providing efflux/resistance when heterologously expressed may appear strange if we consider that the remaining components of the putative efflux pump are missing. One possible explanation for the observed effect is that the periplasmic adaptor proteins (PAPs) of the *E. coli* are able to pair effectively with VcaM providing at least some level of compatibility with TolC. Indeed, the TolC from *Vibrio vulnificus* has been reported to effectively complement the delta *tolC* in *E. coli* suggesting a high level of compatibility of these efflux systems [39]. Furthermore, the analysis of the TolC sequences from *V. cholerae* demonstrate that in particular, VC2436 protein which appears to be the closest TolC homologue in *Vibrio cholerae* O1 shows 47% sequence identity to TolC of *E. coli* K12 (as determined by the SIM expasy server) and displays high level of similarity in the region of periplasmic helical turns which are supposed to be involved in PAP-binding [22]. 

### 2.4. The Efflux of H33342 Conferred by VcaM/TolC is Independent of Secondary Active Transporters in E. coli

To establish whether there is a one-step translocation of VcaM and the OMF TolC, possibly with the help of PAPs, or whether the efflux may be mediated by a secondary transporter different from AcrB as a two-step translocation (e.g., AcrD, AcrF or MdtABC) [40], we performed efflux assays in the presence of the RND-pump-specific inhibitor PAβN (phenylalanine-arginine beta-naphthylamide) and general secondary-transporter inhibitor CCCP (Carbonyl cyanide m-chlorophenylhydrazine) as follows. It was previously reported that the RND transporter AcrAB-TolC complex and its homologue MexAB-OprM may capture substrates from the periplasm, because carbenicillin, which does not diffuse across the cell membrane, was effluxed by the AcrAB-TolC complex [41]. In addition, the RND transporter AcrD can also capture aminoglycosides from the periplasmic space to extrude them into the extracellular space in intact cells [26].

**Figure 4:**
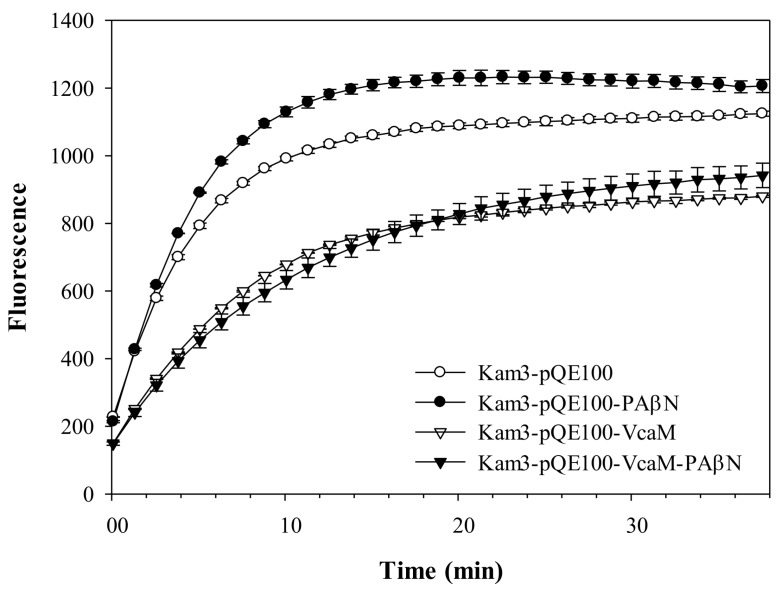
Drug accumulation of H33342 in *E. coli* Kam3 overexpressing VcaM in the presence of RND-transporter inhibitor PAβN. H33342 was incubated for 38 min in the presence of 20 μg/mL PAβN.

**Figure 5:**
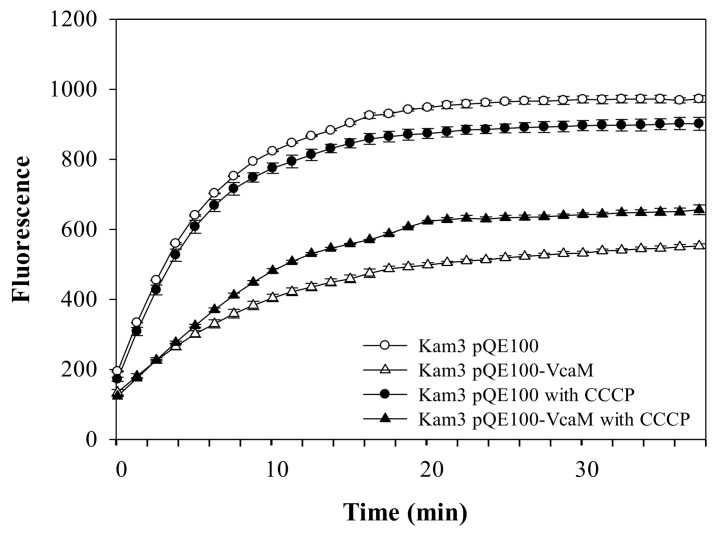
Drug accumulation of H33342 in *E. coli* Kam3 overexpressing VcaM in the presence of membrane decoupling secondary transporter inhibitor CCCP. H33342 was incubated for 38 min in the presence of 20 μg/mL CCCP.

In order to understand whether the H33342 efflux by VcaM/TolC was mediated by secondary active efflux pump systems (two step translocation), the accumulation of H33342 in the Kam3 cells (Δ*acrB*) harbouring a plasmid encoding VcaM (Kam3-pQE-VcaM) in the presence and absence of the RND-pump-specific inhibitor PAβN (Figure 4) and general secondary active transporters inhibitor CCCP (Figure 5) were tested. 

The accumulation of H33342 was lower in the cells expressing VcaM than the cells harbouring empty plasmid. CCCP is a protonophore, which causes dissipation of the membrane potential, thus, affecting all the secondary transporter-dependent efflux [42], and and the peptidomimetic compound PAβN has been shown to be a RND-pump inhibitor [43]. Addition of PAβN and CCCP to the cells expressing VcaM did not abolish H33342 efflux (*p <* 0.05), suggesting that the TolC-dependent efflux of H33342 by VcaM does not require secondary active transporters, such as AcrD and YhiV (MdtF) [44,45]. 

Thus, the translocation of H33342 by VcaM/TolC might be a one-step translocation from cytoplasm to extracellular space.

### 2.5. Dependence on OMF TolC 

**Figure 6:**
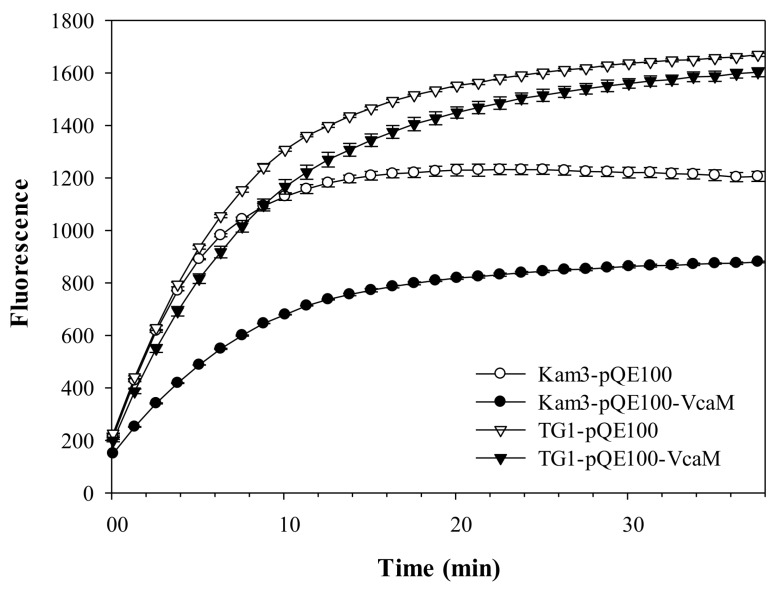
Drug accumulation of H33342 in drug sensitive strain *E. coli* Kam3 and TG1 overexpressing VcaM.

Our previous data indicated that secondary transporters are not required for the function of VcaM, and we, therefore, wanted to confirm that the OMF actually is directly responsible for the effects observed in its heterologous expression. Consistent with the TolC-dependence of the VcaM efflux, the accumulation of H33342 was high (high fluorescence) in the TG1 (Δ*tolC*) cells harbouring empty plasmid and the plasmid encoding VcaM (TG1-pQE-VcaM), indicating VcaM required TolC for H33342 efflux (Figure 6). 

Importantly, and consistent with the interpretation that VcaM is capable of directly engaging with the OMF, the Kam3 cells (Δ*acrB*) harbouring the plasmid encoding VcaM had lower H33342 accumulation as compared the Kam3 cells harbouring empty control plasmid, indicating the simultaneous presence of both VcaM and TolC was sufficient (*p <* 0.05) to confer efflux of the substrate dye H33342 (Figure 6).

**Figure 7:**
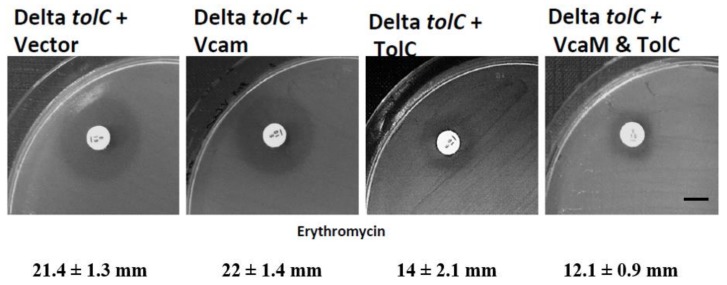
Representative disk diffusion assay results demonstrating the VcaM dependency on TolC. 10 μg impregnated erythromycin disk was placed and incubated at 37 °C overnight.

The disk diffusion assay was performed in the cells lacking the outer membrane factor TolC [*E. coli* BW25113 cells (Δ*tolC*) (DE3)] harbouring control plasmid, the plasmid encoding VcaM, the plasmid encoding TolC, and the plasmid encoding both VcaM and TolC (Figure 7). The diameter of zone of inhibition for the erythromycin (10 μM) disk was measured, the inhibition zone of the BW25113 cells expressing both VcaM and TolC (12.1 ± 0.9 mm) was smaller than the cells harbouring plasmid encoding VcaM (22 ± 1.4 mm) or TolC alone (14 ± 2.1 mm), suggesting that VcaM conferred a high level of resistance to erythromycin when co-expressed with TolC. This data is consistent with our previous result on the efflux of H33342 by VcaM, and its dependency on TolC (Figure 7).

It has been previously reported that TolC was required for the function of ABC-type macrolide transporter MacB, RND transporter AcrB and MFS transporter EmrB, which cooperate with PAPs MacA, AcrA and EmrA and outer membrane channel TolC to form a tripartite efflux system to enhance the resistance of antibiotics [40,46,47]. Moreover, a previous report demonstrated that TolC from *E. coli* could replace VceC from *V. cholerae* to form a functional tripartite efflux system with VceAB-TolC in *E. coli* cells [48], displaying a high degree of OMF-PAP promiscuity.

### 2.6. Periplasmic Adapter Proteins Might be Needed for V. cholerae VcaM to Provide TolC-Dependent Efflux

The VcaM topology prediction [49] indicated that it contains six transmembrane helices (Figure S1) and lacks big periplasmic loops, suggesting it might need a PAP to bridge the OMF form for a one step translocation. In the tripartite pump MacA/MacB/TolC which confers resistance to macrolides, the *macA* gene (Genbank NC_000913, ECK0869) is located next to *macB* gene (Genbank NC_000913, ECK0870) on the *E. coli* K12 chromosome (Genbank NC_000913) [18]. Intriguingly, there is no PAP sequence located next to the *vcaM* gene (Genbank NC_002506.1, VCA0996) in *V. cholerae*. 

Our efflux and disk diffusion assay results indicated that VcaM conferred efflux with the help of *E. coli* TolC, possibly mediated by a PAP in *E. coli* based on their close homology and potential cross-reactivity. Such interpretation is reinforced by previous reports of cross-reactivity and functional substitution between TolC and VceC [48]. Thus, we surveyed the possible drug efflux-associated PAP that might combine with VcaM in *E. coli* along with their homologues in *V. cholerae* (see Table 1 above*)*.

**Figure 8:**
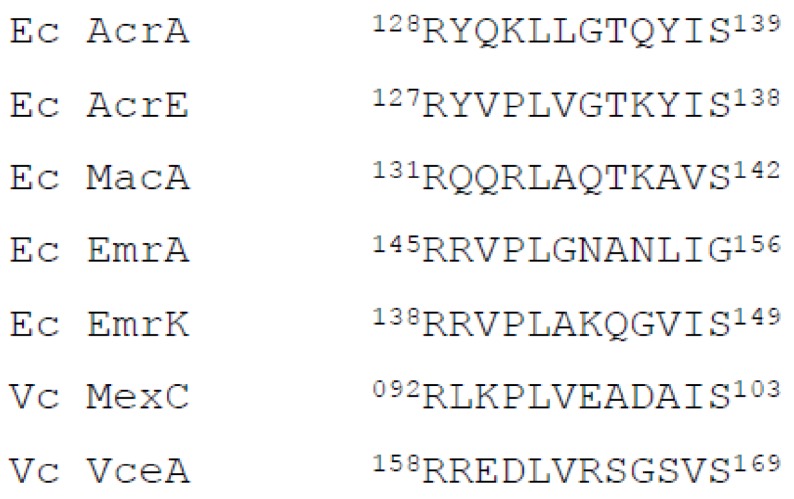
Sequence alignment of the PAPs in *E. coli* and *V. cholerae*. Ec AcrA (Genbank NP_414996); Ec AcrE (Genbank NP_417731); Ec MacA (Genbank NP_415399); Ec EmrA (Genbank NP_417170); Vc MexC (Genbank WP_001131865.1); Vc VceA (Genbank NP_231053).

Moreover, *E. coli* AcrA, AcrE, MacA and EmrK and *V. cholerae* MexC and VceA share a common structural motif RxxxLxxxxxxS (RLS or RLT) (x stands for any residues) (Figure 8) [50,51], which is believed to play an important role in the interaction with the TolC aperture and can provide an explanation for such a promiscuity of interaction between heterologous PAPs and OMFs. Our data suggests that the recombinant *V. cholerae* VcaM is able to interact with the endogenous *E. coli* TolC and PAPs for one-step translocation of substrates, which might reflect the existence of a similar mechanism in *V. cholerae*, however, further work is required to determine the exact PAP with which it engages in situ. 

While ABC-transporters of the HlyB family are well-known to associate with TolC channels forming part of the tripartite Type I secretion systems (T1SS) [51,52], the only ABC-drug transporter with established TolC dependency is the MacB. MacB, however, presents a unique architecture amongst the ABC transporters [29,35,53] by possessing a pronounced periplasmic domain with structural similarities to the larger RND transporters [29,54], allowing it to assemble PAP proteins containing membrane-proximal domains (MPDs) and recruit TolC [55]. Notably, no classical 6TM-spanning dimeric ABC-transporters that seem to share the closest similarity with VcaM i.e., Sav1866 or MsbA-like families have been demonstrated to be able to associate with TolC to date. It is, however, conceivable that the arrangement within a putative tripartite system containing VcaM is similar to the one within the T1SS-associated transporters (which also share the predicted VcaM topology of being dimers with 6TM-per subunit) or MFS-transporter-based assemblies [52,56]. Both of these require PAP-proteins lacking MPDs to effectively seal the tripartite assembly, which are not readily identifiable in the *Vibrio* genome. As we have discussed above, cross-reactivity of VcaM with endogenous PAPs within *E. coli* could possibly provide an explanation for the TolC-dependent function observed in a heterologous expression background. Indeed, a sequence analysis of the *Vibrio* genome reveals at least five TolC orthologues which demonstrates a wide variety of potential pairings for the VcaM, which some of these OMFs exhibiting high-level of similarity (over 70%) with the *E.coli* TolC [22]. 

While future research is required to elucidate the exact mechanism by which the VcaM-family of ABC-transporters engage TolC, our clear demonstration of its TolC-dependency is both important and intriguing, as it makes it the only currently known member of the ABC-family lacking pronounced periplasmic domains that is capable of recruiting TolC while having an established drug-efflux function. 

## 3. Materials and Methods

### 3.1. Bacterial Strains, Media and Chemicals

The *vcaM* gene was from *V. cholerae* strain CVD101 (CT^–^A^–^, CT^–^B^+^ deletion derivative of classical biotype Ogawa serotype 395) chromosomal DNA [57]. *Escherichia coli* C43 (DE3) were used for protein expression, *Escherichia coli* Kam3 (DE3) which is the *acrB* deletion strain and *E. coli* TG1 Δ*tolC* (a derivative of TG1 that lacks *tolC*) were used in drug accumulation assay [58]. *E. coli* BW25113 Δ*tolC* was converted to (DE3) by DE3 lysogenization (Novagen) and used for disk diffusion assays. The bacteria were grown in Luria–Bertani broth (LB), 2XYT medium broth and Mueller-Hinton broth (MH broth). The ampicillin (100 μg/mL) was used in the experiment.

### 3.2. Cloning of VcaM

*vcaM* gene was cloned from the *V. cholerae* CVD101 chromosome by using PCR method. The *vcaM* gene amplified by using primers 5′-GGGGGGCATATGTTTAAGCTATTTGAAGGTTTCACCGAC-3′ and 5′-TTTTTTCTCGAGTGCCAGTATGGCTTCTTCTAC-3′ which was digested with *Nde*I and *Xho*I restriction enzymes and inserted onto pET21a vector at the *Nde*I-*Xho*I site. The *vcaM* gene was amplified using the primers, the PCR products ere digested with *Bam*I and *Pst*I restriction enzymes and inserted onto pQE100 vector at the *Bam*I-*Pst*I site. The pET21a plasmid encoding VacM was transform into *E. coli* C43 (DE3) for protein overexpression and the pQE100 plasmid encoding VcaM was transform into *E. coli* Kam3 and TG1 Δ*tolC* for drug susceptibility test. *E. coli* tolC was amplified from *E. coli* K12 strain with forward and reverse primer 5′-AATAACATATGAAGAAATTGCTCCCCATTCTTATCGGC-3′ and 5′-AATAACTCGAGGTTACGGAAAGGGTTATGACCGTTACTGG-3′. The PCR products were digested with *Nde*I and *Xho*I restriction enzymes and cloned into pET21a vector.

### 3.3. Expression and Purification

*E. coli* C43 (DE3) harboring plasmid encoding VcaM was cultivated at 37 °C, induced with 0.2 mM IPTG and overexpressed at 24 °C. The cells were collected by centrifugation and disrupted by using French press. The pellet was removed by centrifugation, and the supernatant collected. The supernatant was centrifuged at 43,000 rpm for 60 min to spin down the cell membranes, which were collected and solubilised in 2% (*v*/*v*) Triton X-100 (BioShop, Burlington, ON, Canada). Detergent solubilized VcaM was purified by using nickel affinity column (Hitrap chelating column, GE, Boston, MA, USA) [59].

### 3.4. SDS-PAGE and Western Blot

Recombinant VcaM was resolved by SDS-PAGE to determine the efficiency of overexpression and purification. As for western blot, VcaM samples were run using two SDS-PAGE gels by gel electrophoresis in duplicate, applied as experimental gel and control gel. The proteins on the experimental gel were transferred to a PVDF a PVDF (Polyvinydene fluoride) membrane using semi-dry transfer (175 mA, 75 min). The membrane was washed three times with wash buffer (7.5 mM K_2_HPO_4_, 150 mM NaCl, 0.05% (*v*/*v*) Tween 20, pH 7.4) and saturated with blocking buffer (wash buffer containing 3% bovine serum albumin) at 4 °C. After three washes with wash buffer, the membrane was incubated with primary antibody (mouse anti-histidine tag) for 1 h. The PVDF membrane was then incubated with a goat anti-mouse IgG-AP (alkaline phosphatase) conjugate as a secondary antibody, and the proteins were identified by using a colorimetric AP conjugate substrate kit (Bio-Rad, Hercules, CA, USA) [18].

### 3.5. ATPase Activity Assay

The ATPase activity was measured by malachite green assay as described previously [60]. The reaction buffer containing 20 mM Tris (pH 7.4), 200 mM NaCl, 10 mM MgCl_2_, 0.2% Triton X-100 and 0.5 μM VcaM was mixed with adenosine 5′-triphosphate disodium salt (ATP) and incubated at 37 °C. 5 mM MgCl_2_ was added to initiate ATPase activity, and sample was taken from the reaction mixture to mix with EDTA (Ethylenediaminetetraacetic acid)to stop reaction on a time point from 0 to 10 min. Malachite green solution was added to each sample and an absorbance at 610 nm was measured by a spectrophotometer [60]. The rate of ATP hydrolysis was measured as a function of the ATP concentration and the data fitted to a sigmoidal equation using SigmaPlot (Systat Software Inc. San Jose, CA, USA)).

### 3.6. Hoechst (H) 33342 Accumulation Assay

The H33342 bis-benzamide accumulation assay was carried out as previously described [61], with the following modifications. The *E. coli* cells were grown to OD_600_ of 0.6 to 0.8 in MH broth and collected by centrifugation (5000 rpm, 5min and 4 °C). The cells were resuspended twice in PBS buffer and diluted in PBS in a final OD_600_ of 0.6. The cell suspension was incubated in 96 well plate with the filter-sterilized glucose to a final concentration of 25 mM at room temperature for 3 min. The H33342 dye was added to a final concentration of 1 μM and the fluorescence was measured over 38 min at excitation and emission wavelengths of 360 and 460 nm [61]. The efflux pump inhibitors of NaOV, CCCP and PAβN was added to a final concentration of 40 μg/mL, 20 μg/mL and 20 μg/mL, respectively.

### 3.7. Disk Diffusion Assay

The disk diffusion assay was performed as previously described [62] with slight modification. *E. coli* BW25113 Δ*tolC* cells were transformed with pACYC *tolC* from *E. coli* and or pET21a *vcaM* from *V. cholerae*. The fresh transformants were grown in LB media supplemented with respective antibiotics to an OD_600_ of 0.5, induced with 0.25 mM IPTG for 2 h at 37 °C. The induced cells were diluted to an OD_600_ of 0.1 and spread the cells using sterile cotton swabs on Muller Hinton Agar (MHA) incorporated with 0.25 mM IPTG. The plated were allowed to dry for a minute, 10 μg impregnated Erythromycin disk (Oxoid) was placed and incubated at 37 °C overnight. Then the diameter of zone of inhibition along the disk was measured and recorded. The assay was performed in triplicates.

### 3.8. Prediction of Transmembrane Regions

The transmembrane regions of VcaM was predicted by using TMHMM server (Available online: http://www.cbs.dtu.dk/services/TMHMM/) [49].

### 3.9. Protein Sequence Alignments

The protein sequences of MexC (GenBank WP_001131865.1) and VceA (GenBank NP_231053) from *V. cholerae*, AcrA (GenBank NP_414996)/AcrE (GenBank NP_417731)/MacA (GenBank NP_415399)/EmrA (GenBank NP_417170) and EmrK (GenBank D78168) from *E. coli* were obtained from the National Center for Biotechnology Information (NCBI, available online: www.ncbi.nlm.nih.gov), and the sequences were aligned by using Clustal Omega multiple sequence alignment (Hinxton, Cambridge, UK) [63].

### 3.10. Statistical Analysis

Data were statistically analyzed using SPSS Version 12.0 (SPSS Inc., Chicago, IL, USA). One-way analysis of variance (ANOVA) was used to determine the statistical differences between the sample means, with the level of significance set at *p* < 0.05. Multiple comparisons of the means were conducted using the Tukey test. All data are expressed as mean ± SD.

## 4. Conclusions

In this study, we confirmed VcaM as a bona-fide drug ABC-transporter by giving the detailed kinetic parameters of ATP hydrolysis, and reported VcaM as the only multidrug ABC-transporter outside of the MacB-family with demonstrable TolC-dependency. A TolC-dependent efflux by VcaM was observed in dye accumulation and disk diffusion assays. The secondary active transporter inhibitors, PAβN and CCCP, did not retard the efflux of H33342 conferred by VcaM, indicating that VcaM might confer a one-step translocation in a TolC-dependent manner. A periplasmic adapter protein could be involved to link VcaM and TolC in the construction of a tripartite pump for substrate translocation, although future research is clearly required to elucidate the exact mechanism. 

## Figures and Tables

**Table 1:** *E. coli* PAP homologues in *V. cholerae*.

PAPs	Inner Membrane Transporter	PAP Homologues in *V. cholera* (SEQUENCE Identity %)	Reference
RND family			
AcrA	AcrB; AcrD	MexC, 36%	[26,27]
AcrE	AcrF	MexC, 36%	[28]
ABC family			
MacA	MacB	Hemolysin D, 38%	[29]
MFS family			
EmrA	EmrB	VceA, 39%	[17,30]
EmrK	EmrY	VceA, 40%	[30,31]

PAPs, periplasmic asapter proteins; RND, Resistance-Nodulation-Division; MFS, Major Facilitator Super family; AcrA (Genbank NP_414996); AcrB (Genbank NP_414995); AcrD (Genbank NP_416965) MexC (Genbank WP_001131865.1); AcrE (Genbank NP_417731); AcrF (Genbank NP_417732); MacA (Genbank NP_415399); MacB (Genbank NP_415400); Hemolysin D (Genbank WP_000083084.1); EmrA (Genbank NP_417170); EmrB (Genbank NP_417171); VceA (Genbank NP_231053); EmrK (Genbank NP_416869); EmrY (Genbank NP_416868).

## Supplementary Materials

The following are available online at http://www.mdpi.com/1422-0067/19/4/1000/s1.
