# Supplementary material for: ATP-Binding Cassette Transporter VcaM from Vibrio cholerae is Dependent on the Outer Membrane Factor Family for Its Function
Source: Int J Mol Sci. 2018 Mar 27;19(4):1000. doi: 10.3390/ijms19041000 (PMC5979437; doi:10.3390/ijms19041000)
Supplement: Supplementary file 1 [file ijms-19-01000-s001.pdf]

001 MFKLFEGFTD PFPKGEPQRP SNTLWAFCRH YTHGFEKPLI VMALLSTAIA 050

051 IIEVSLFGFM GQLVDWLSTS SPDTFLVENQ STLIGLGLLV LVGMPLLIAF 100

101 YSLLIHQSLL GNYPMSIRWL AHRYLLKQSV SFYQDEFAGR ISTKVMQTAL 150

151 AVRETVMKSL DVFVYVMVYF TAIVVILAQA DWRLMIPMLI WLAIYVTVQM 200

201 YYVPKLKKVA SEQADARSLM SGRIVDSYTN IMTVKLFSHS QRETQYAEEG 250

251 MQDFLGTVHR QMRLVTGFNI WVEMANYLLV FTIAALSIYL WTTSAISVGA 300

301 IAVAVSLSLC INGMSKWIMW EVSALFENIG TVVDGMTMLG KPITVTDKPD 350

351 AKPLVVKHGG ITFDDVSFHY GENKSVINHL NLNIKPGEKV GLVGRSGAGK 400

401 STLVNLLLRF HDVESGRILI DGQPISEVTQ ESLRSKIGMV TQDTSLLHRS 450

451 IRDNILYGNP NATEEQLLKA TAQAHAHEFI LGLTDPHGNS GYDAQVGERG 500

501 VKLSGGQRQR IAISRVLLKD APLLVLDEAT SALDSEVEAA IQESLNELMQ 550

551 GKTVIAIAHR LSTIAAMDRL IVLDKGQIVE QGSHQELIAQ NGIYAHLWAH 600

601 QTGGFIGCDE DEVEEAILA 619

S1 Figure. The predicted transmembrane regions of VcaM from *V. cholerae* (Genbank, Q93GU0). The estimated transmembrane helices amino acid residues: 39–61, 83–105, 162–180, 184–203, 264–286, 290–312 amino acid residues, predicted by TMHMM.
